# Alpha-fetoprotein level in fetuses, infants, and children with ovarian masses: a literature review

**DOI:** 10.3389/fendo.2024.1307619

**Published:** 2024-02-06

**Authors:** Aleksandra Matonóg, Agnieszka Drosdzol-Cop

**Affiliations:** Department of Gynecology, Obstetrics and Oncological Gynecology, Faculty of Health Sciences inKatowice, Medical University of Silesia, Katowice, Poland

**Keywords:** alpha-fetoprotein, ovarian masses, fetal ovarian cyst, germ cell tumors, reference value

## Abstract

Alpha-fetoprotein (AFP) is a serum protein highly produced during the fetal period. It is also known as a biomarker of various pathologies. Commonly, tumors requiring diagnosis and monitoring through AFP determination appear during the first year of life, with poorer outcomes when presenting in fetal life. Due to advancements in imaging technology, the detectability of ovarian masses in infants is higher. However, the use of AFP as a biomarker could improve diagnosis in cases when imaging and histological examinations are not sensitive enough to detect tumors. From the outcome of our investigation, it is possible to conclude that there is evidence of an association between increased AFP levels and ovarian masses. However, previous studies have presented contradictory and unverified results, with the authors emphasizing that future research is needed. In this article, an analysis of the available literature on AFP as a biomarker of ovarian masses in children was performed. Two types of literature were reviewed: guidance and published studies (clinical trials, reviews, and systematic reviews). We searched the Embase, PubMed, ScienceDirect, and Web of Science databases to collect essential data.

## Introduction

Alpha-fetoprotein (AFP) plays a significant role in fetal life, but its concentrations decline during the first year of life and remain physiologically at low levels over the entire lifetime. In many studies, confirmation of AFP as a useful marker of various pathologies could be found. In clinical practice, AFP is used as a tumor and gestational marker; however, more and more research studies have indicated a relationship between AFP levels and ovarian masses (1, 2).

Ovarian lesions are not as uncommon in children as it might seem. In the prenatal period, the most common ovarian masses are fetal ovarian cysts. Previous studies have shown ovarian cysts to have an annual incidence of 1 case per 2,500 live births. Nevertheless, there continues to be a lack of global guidelines, uniform diagnosis, and treatment measures. The etiology of this pathology is still unknown, albeit most researchers have considered hormonal stimulation. Importantly, children diagnosed with fetal ovarian masses generally have a good prognosis; however, awareness is required in cases with acute abdominal pain, suggesting severe conditions such as ovarian torsion. There appears an opportunity to improve the diagnostics through the use of AFP expression. However, interpretation of the levels of AFP is difficult due to the lack of a normal laboratory range for this protein (1, 3).

## Alpha-fetoprotein

AFP is a mammalian glycoprotein that was isolated for the first time by C. Bergstrand and B. Czar in 1956 from the serum of human fetuses. This plasma protein is a 70-kDa single polypeptide encoded by the *AFP* gene on chromosome 4q25. The half-life of AFP is 4–5 days. Its electrophoretic profile revealed that it occupies an α-1 anodic position, running slower than albumin (4).

The level of AFP is increased from approximately the 14th week of gestation until it falls from the 32nd week of pregnancy. Its levels in the blood serum of term and healthy infants can reach 50,000 ng/ml. The AFP level is notably elevated in preterm newborns due to liver and intestinal hyperplasia. Some authors have proposed that a low birth weight is the cause of increased AFP levels. Interestingly, in both groups (preterm and term infants), a biphasic pattern on a semi-logarithmic plot has been described. This consists of, firstly, a rapid reduction of the AFP level followed by a slower decrease (1, 4).

AFP is produced mainly by the fetal liver and the embryonic yolk sac. Trace amounts of AFP are synthesized in the kidneys and the gastrointestinal tract of the fetus. Moreover, it could also be re-expressed in tumors during adulthood. In many studies, confirmation of AFP being a useful marker of various pathologies could be found. Measurement of the AFP concentrations in the serum of children is recommended for the diagnosis and treatment of, e.g., hepatoblastoma, hepatocellular carcinoma, and germ cell tumors (4–6).

The AFP level is also a commonly known predictor of pregnancy complications, e.g., preeclampsia, intrauterine growth restriction (IUGR), and placental abruption. In addition, triple and quadruple screening tests contain maternal serum AFP as a marker of fetal pathologies. High AFP levels in maternal serum are associated with neural tube defects (e.g., spina bifida), omphalocele, gastroschisis, sacrococcygeal teratoma, placental and renal abnormalities, cystic hygroma, osteogenesis imperfecta, threatened abortion, IUGR, and decreased maternal weigh. Lower levels occur in trisomy 21, trisomy 18, and increased maternal weight (4, 7).

Regarding interfering factors, which could be implicated in false-positive results, multiple gestation and gestational diabetes are included. Interestingly, the level of maternal serum AFP is a little higher in smokers in singleton pregnancy (approximately 7%, which is a statistically significant increase). However, such a correlation has not been observed in twin pregnancy (4, 8).

## Relationship between α-fetoprotein level and ovarian masses

As studies have shown, the level of AFP can also be used as a marker of tumorous lesions of the ovary, and it is also useful in treatment response assessment and recurrence monitoring. The AFP level is a reliable marker of primary vaginal endodermal sinus tumor in infants and children. The level of this glycoprotein is elevated in cases of malignant germ cell tumors, choriocarcinoma, and embryonal cell carcinomas. It is estimated that approximately 69% of yolk sac tumors occur in groups of neonates. Unfortunately, they often coexist with teratomas, in which case they are characterized by poor prognosis and high mortality (1, 3–5).

The most significant finding in this paper was that of a recent study by Li et al. (2021), which showed that the serum AFP level was elevated among newborns with ovarian cysts. This result remains briefly addressed in the literature. The lack of a standardized normal range for serum AFP, depending on the age of the child and the birth weight, creates a serious diagnostic problem (1, 3).

### Ovarian masses in infants

The etiology of ovarian masses is unclear; however, diverse hypotheses exist. Most of these regard the influence of various hormones on the fetal ovary, such as fetal gonadotropins, maternal estrogen, and placental human chorionic gonadotrophin. Intra-abdominal masses are infrequent in female fetuses although the most common source comprises the ovary. The majority of them originate from follicular epithelial tissues, but there are also theca lutein and corpus luteum cysts, or simple cysts, the origins of which are not defined. Interestingly, ovarian masses tend to be self-limiting; therefore, most can resolve spontaneously during the first months of a child’s life. This is caused by the reduction of maternal hormone stimulation. The occurrence of ovarian cysts is not a life-threatening condition; nevertheless, there is a risk of ovarian torsion, which is a complication that is definitely dangerous to patients. Moreover, there is a possibility of the progression of simple cysts to complex cysts, which increases the risk of ovarian loss (9–11).

Fortunately, due to medical advances, ovarian masses can be diagnosed earlier. The most common type of abdominal tumor diagnosed in the prenatal period is ovarian cysts. Particularly, in a group of girls younger than 2 years, benign or malignant ovarian tumors, in rare cases, have been observed. Studies have shown that the incidence of fetal ovarian cysts is 1:2,500. In children aged over 10 years, ovarian tumors are more common. Ovarian cysts are mostly unilateral and diagnosed in the last trimester. Unfortunately, there is still the problem of the lack of uniform diagnosis and treatment measures (3, 9, 12).

In accordance with the literature, the probability of ovarian cysts increases in children of mothers who suffer from diabetes mellitus, Rh isoimmunization, and preeclampsia. This is probably due to the fact that these conditions are related to excess fetal gonadotropins. However, Min et al. (2022) collected data on children diagnosed with ovarian masses during prenatal development and showed that their occurrence is not related to multiple pregnancies or abnormalities during pregnancy (e.g., gestational diabetes and eclampsia) (10, 12).

Early termination of pregnancy should be performed to preserve ovarian function, due to which postnatal treatment is done earlier. However, several studies have indicated that fetal ovarian cysts sometimes shrink spontaneously during pregnancy, and early termination of pregnancy can result in an increase in the number of unnecessary surgical procedures. It is worth underlining that a higher rate of ovarian loss was noted in children diagnosed prenatally with a simple ovarian cyst, which became complex in the postnatal period (9, 11).

### Ovarian torsion

Importantly, it should not be forgotten that the increased weight of the ovary caused by masses could lead to ovarian torsion, especially when it is larger than 5 cm. This severe condition could lead to hemorrhagic infarction and necrosis of the ovarian stroma. In infants, ovarian torsion is mainly caused by cysts, whereas in childhood it is frequently associated with ovarian mature teratoma. Although it is a rare condition in infants and children, ovarian torsion should always be considered in cases of severe lower abdominal pain, especially in combination with fever, nausea, and vomiting. However, the clinical presentation of such a condition in newborns could be often more unobvious and underlying than that in older children. The diagnosis of ovarian torsion in children is predominantly grounded on ultrasound, rarely on computed tomography (3, 5).

### Reference values for AFP

Reliable data on the reference values for AFP are not available. Previous studies presented contradictory and incomplete results. The only available figures are from 20 years ago, for which the research methods are questionable. In these research studies, a small series of infants were enrolled. Furthermore, various mathematical and statistical methods were used, which meant that the results could not be appropriately interpreted (13).

Bader et al. (2004) conducted a large literature review and attempted to determine the AFP serum level in the early neonatal period. According to their research, the reference interval for AFP concentrations at birth was 15.7–146.5 Ag/ml, which was based on a 95% confidence interval (CI). They found no significant differences between female and male neonates. However, the authors emphasized that future research is needed because of the wide range of normal values. The study of serum AFP concentration in extremely low-birthweight infants (ELBWI) was first carried out by Maruyama in 2017. In general, the results showed a correlation between the serum AFP concentration and age in ELBWI. However, only 23 infants were included in the study (13, 14).

In the future, we would like to determine the normal range of serum AFP depending on the age of the child and with plotting a logarithmic curve in a group of infants.

## Discussion

Based on the analyzed literature on ovarian masses and their relationship with AFP serum level, a prospect to improve the diagnosis could be found. Potentially, assessment of the AFP level can contribute to the earlier detection of fetal ovarian cysts and increase the chances of preserving ovarian function. The available upper reference limits for AFP have been considered by researchers as unreliable and unsuitable. Moreover, hitherto, the concentration curve of AFP has not been determined. More research into this topic is still necessary, especially concerning the normal range of AFP serum depending on the age of the child. Plotting a logarithmic curve in a group of infants is also required due to the incidence of ovarian lesions in this group being very high. These steps could improve the diagnosis when imaging and histological examinations are not sensitive enough to detect the tumor (1).

## Author contributions

AM: Conceptualization, Data curation, Formal analysis, Project administration, Writing – review & editing. AD-C: Project administration, Supervision, Writing – review & editing.
